# Editorial: Beclin 1 and autophagy---in memory of Beth Levine (1960–2020)

**DOI:** 10.3389/fcell.2022.1058861

**Published:** 2022-10-24

**Authors:** Haidong Xu, Zheng-Hong Qin, Yongjie Wei, Junchao Wu

**Affiliations:** ^1^ Department of Pharmacology and Laboratory of Aging and Nervous Diseases, Jiangsu Key Laboratory of Neuropsychiatric Diseases, College of Pharmaceutical Sciences, Soochow University, Suzhou, China; ^2^ Cancer Hospital and Research Institute, Guangzhou Medical University, Guangzhou, China

**Keywords:** Beclin 1, autophagy, Beth Levine, cell survival and death, memoriam

Autophagy is an ancient pathway by which parts of cytoplasmic materials are delivered to the lysosome for degradation. Beclin 1, a human ortholog of yeast Atg6/Vps30, is the first mammalian autophagy gene discovered by Beth Levine in 1999 (Aita et al., 1999; Liang et al., 1999). The discovery of Beclin 1 starts a new era of autophagy. From then on, many landmark studies of Beclin 1 from the Beth lab elucidate various functions and roles of autophagy in cell survival, longevity, innate immunity, and cancer. For example, the Levine lab identify the interaction of Beclin 1 with anti-apoptotic protein Bcl-2, which inhibites Beclin 1-dependent autophagy (Liang et al., 1998; Pattingre et al., 2005). They also find that Beclin 1 is essential for lifespan extension in *C. elegans* (Melendez et al., 2003) and the disruption of the association of Beclin 1 and Bcl-2 promotes the lifespan extension in mice (Fernandez et al., 2018). Besides, Beth make many key findings about viral disease (Orvedahl et al., 2010), discovers Beclin 2 (He et al., 2013), and coins the term xonophagy (Levine, 2005). Those exciting findings infuse the autophagy research field and make Beclin 1 the most extensively studied molecule in this field. Discovery of the new functions or modifications of Beclin 1 along with the identification of a series of Beclin 1 interacting proteins significantly improved our knowledge about autophagy initiation and regulation, as well as its essential roles in many human diseases. The breakthrough of Beclin 1-related studies also contributes to the final recognition by the Noble Committee and the awarding of the 2016 Noble Prize in Physiology or Medicine for research in autophagy. Beth Levine, an admirable pioneer in the field of autophagy, passed away on 15 June 2020, after a long-fought with breast cancer. The Chinese poetry says: “The deceased has gone with the Yellow Crane, and only left the empty Yellow Crane Tower here.” Through collecting the reminiscence articles and the studies of Beclin 1 or autophagy related articles, this Research Topic aimed to commemorate Beth Levine for her tremendous contribution and indelible impact on the autophagy field. This Research Topic includes a review about Beth Levine’s legacy written by the mentees from the Beth lab, two original research articles on Beclin 1/autophagy and an opinion about reticulophagy, a form of autophagy selective degradation of the endoplasmic reticulum. Sano et al. show that neutrophil extracellular traps (NETs) promote atherosclerosis by regulating the activity of autophagy in macrophages. They find that NETs upregulate EGFR activity, enhance Beclin 1 phosphorylation of tyrosine residues of Beclin 1 by EGFR, thus inhibite the PI3 kinase activity and autophagosome formation. Vega-Rubin-de-Celis et al. have all worked with Dr. Beth Levine in her lab before. In their review, they introduce some of the discoveries made by the group of Beth Levine and hope to honor her legacy in science.

Beclin 1 exerts autophagy functions mostly through the activation of various Beclin 1-binding proteins. One of the challenges is to elucidate the precise roles of those increasingly identified interacting candidates in autophagy under different cellular or tissue contexts. Another question is that how to fine tune autophagy through manipulating Beclin 1 and use it for clinical treatments. Recently, the Levine lab identify a candidate therapeutic autophagy-inducing peptide, Tat-BECN1 (Shoji-Kawata et al., 2013). The potential mechanism and long-term effects of this peptide still need further study.
